# Py_3_-FITC: a new fluorescent probe for live cell imaging of collagen-rich tissues and ionocytes

**DOI:** 10.1098/rsob.200241

**Published:** 2021-02-10

**Authors:** Zhaotong Wang, Takamasa Mizoguchi, Takahito Kuribara, Masaya Nakajima, Mayuu Iwata, Yuka Sakamoto, Hiroyuki Nakamura, Toshihiko Murayama, Tetsuhiro Nemoto, Motoyuki Itoh

**Affiliations:** Graduate School of Pharmaceutical Sciences, Chiba University, Japan

**Keywords:** live imaging, polypyrrole, collagen-rich tissue, ionocyte, zebrafish

## Abstract

Polypyrrole-based polyamides are used as sequence-specific DNA probes. However, their cellular uptake and distribution are affected by several factors and have not been extensively studied *in vivo*. Here, we generated a series of fluorescence-conjugated polypyrrole compounds and examined their cellular distribution using live zebrafish and cultured human cells. Among the evaluated compounds, Py_3_-FITC was able to visualize collagen-rich tissues, such as the jaw cartilage, opercle and bulbus arteriosus, in early-stage living zebrafish embryos. Then, we stained cultured human cells with Py_3_-FITC and found that the staining became more intense as the amount of collagen was increased. In addition, Py_3_-FITC-stained HR cells, which represent a type of ionocyte on the body surface of living zebrafish embryos. Py_3_-FITC has low toxicity, and collagen-rich tissues and ionocytes can be visualized when soaked in Py_3_-FITC solution. Therefore, Py_3_-FITC may be a useful live imaging tool for detecting changes in collagen-rich tissue and ionocytes, including their mammalian analogues, during both normal development and disease progression.

## Graphical abstract

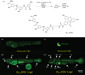

## 1. Introduction

Distamycin is a small antibiotic molecule containing tri-N-methylpyrrole (Py) that binds to the AT-rich DNA minor groove [1]. Poly-Py-based polyamides are used as sequence-specific DNA probes [2]. Therefore, fluorescence-conjugated poly-Py-based polyamides are thought to be useful as nuclear-specific probes. However, several factors influence the nuclear uptake of poly-Py-based polyamides [3]. Best *et al*. [3] demonstrated that poly-Py-based polyamides with good nuclear uptake affinity have several principal features, such as an eight-ring polyamide DNA-binding domain, one or more positive charges, and a conjugated fluorescein fluorophore. Long-length poly-Py-based polyamides are not always effective. Inversely, short polyamides could bind multiple genomic regions [4]. The addition or deletion of a single charge might change the nuclear uptake effect [5]. A moderate length of fluorophore polyamide has many advantages: increasing stabilization, increasing binding intensity, cell nuclear uptake and protein affinity. Therefore, the localization of fluorescence-conjugated polypyrroles in living animals is unclear. We used three types of FITC-conjugated compounds to assess the effect of pyrrole number on their localization in living animals.

Collagen accounts for a large proportion of body mass and is the most common component of the extracellular matrix [6]. Multiple types of collagen play essential roles in body structure. In animals, triple helices assembled in individual collagen molecules create the sophisticated, hierarchical structure that forms the fibres and networks observed in tissue, bone and basement membranes [7]. Furthermore, collagen is involved in diseases; for example, fibrosis is a disease process involving the destruction of healthy tissue through the deposition of a collagen-rich extracellular matrix [8]. Therefore, visualizing collagen localization or dynamics provides essential information for basic studies or early disease diagnosis. Several methods for visualizing collagen have been developed. Second harmonic generation (SHG) imaging can be used to visualize unstained collagen [9,10]. However, a specific device is needed for SHG imaging. Using collagen-binding peptides or proteins, peptide- or protein-based fluorescence probes have been generated [11,12]. These probes are used for staining living cells and dissected living tissue, and can be intravenously injected to detect fibrotic organs. Nevertheless, these high-molecular-weight probes may not be suitable for the whole-body imaging of living animals. A low-molecular-weight probe, Col-F, has been developed [13]. However, because it is a fluorescein-conjugated physostigmine, an inhibitor of acetylcholinesterase, its side effect potential needs to be determined before it can be applied to living animals. A transgenic strategy has also been used for imaging collagen [14]. However, making transgenic lines takes a long time, and only a specific type of collagen can be labelled this way. Low-molecular-weight, low-toxicity-inducing and pan-collagen imaging probes would have advantages for general use in collagen imaging.

In zebrafish (*Danio rerio*), ionocytes transport ions through respective sets of ion transporters and control body osmotic pressure. In zebrafish embryos, several types of ionocytes, such as H^+^-ATPase-rich (HR) cells, Na^+^-K^+^-ATPase-rich (NaR) cells, Na^+^-Cl^−^ cotransporter (NCC) cells, solute carrier 26 (SLC26) cells and K^+^-secreting (KS) cells, are in the skin and gill [15]. HR cells secrete acid, take up Na^+^ and excrete NH_4_^+^ and are similar to mammalian renal proximal tubular cells [15]. Therefore, an easy imaging tool for HR cells may make these cells useful as model systems of mammalian renal proximal tubular cells.

Mitochondrial staining reagents, including MitoTracker, can stain HR cells and NaR cells [16]. Sodium green is a Na^+^-dependent fluorescent reagent that can detect HR cells [17]. However, sodium green is used only in living cells and is not applied to fixed samples, such as immunostaining. To our knowledge, there is no specific and useful probe for HR cells.

Here, we show that newly generated MeO-Py3-GABA-FITC (Py_3_-FITC) can be used to detect collagen-rich tissue in living zebrafish embryos. Py_3_-FITC can be used to visualize cartilage, notochord, fin rays, etc. In addition, Py_3_-FITC also stains HR cells. Py_3_-FITC can be used for double staining with Alizarin Red S or antibodies. These data indicate that our newly developed Py_3_-FITC probe is a powerful tool for imaging collagen-rich tissue and HR cells.

## 2. Methods

### 2.1. Zebrafish

AB (wild type), *Tg (fli:dsRed);casper* [18,19], and *mib^ta52b^*-mutant zebrafish lines were used. The *casper* zebrafish [19] were obtained from the Zon laboratory. *Tg (fli:dsRed); casper* zebrafish were generated by crossing *Tg (fli:dsRed)* with *casper* zebrafish. The *mib^ta52b^* mutant was described previously [20]. The zebrafish were raised and maintained under standard conditions [21] with approval by the Institutional Animal Care and Use Committee at Chiba University (nos 1-174, 2-178). Males and females of these strains were mated to generate embryos.

### 2.2. Experimental methods for synthesis

All reactions were performed with dry solvents under argon, and the reagents were purified by the usual methods. Column chromatography purification was performed with silica gel 60 N (spherical, neutral 63–210 mesh). Preparative thin-layer chromatography (PTLC) separations were carried out on 2 mm E. Merck silica gel plates (60F-254). Nuclear magnetic resonance (NMR) spectra were recorded on JEOL-JMN-ECS400 and ECZ400 spectrometers operating at 400 MHz to obtain ^1^H-NMR spectra. Data from the ^1^H-NMR analysis were reported as follows: chemical shift (*δ* ppm), multiplicity (s = singlet, br-s = broad singlet, d = doublet, t = triplet and m = multiplet), coupling constants (Hz) and integration. High-resolution mass spectra were measured on a JEOL AccuTOF LC-plus JMS-T100 LP spectrometer (ionization method: electronic supplementary material).

### 2.3. Synthesis of 5-FITC-labelled benzylamine

5-FITC-labelled benzylamine was synthesized according to a previously reported procedure [22].

### 2.4. Experimental procedure for the synthesis of MeO-Pyn-GABA-FITC

DIPEA (50 µl, 0.29 mmol, 3.0 eq) was added to a stirred solution of *N*-Boc-4-amino butanoic acid (39.4 mg, 0.19 mmol and 2.0 eq) and HATU (73.8 mg, 0.19 mmol, 2.0 eq) in N,N-dimethylformamide (DMF) (0.24 ml) at room temperature. After stirring for 1 h, the reaction was added to a solution of MeO-Py_3_-NH_2_ (38.6 mg, 0.097 mmol, 1.0 eq) [23], and DIPEA (33 µl, 0.19 mmol, 2.0 eq) was added to DMF (0.24 ml) and stirred at the same temperature for 22 h. Then, the reaction was quenched with water (2 ml) and extracted with EtOAc (2 ml × 3). The combined organic layers were washed with water (2 ml × 3) and brine (2 ml), dried over Na_2_SO_4_, and concentrated *in vacuo*. The crude product was roughly purified by column chromatography eluted with ethyl acetate. TFA (0.11 ml, 1.4 mmol, 20.0 eq) was added to a stirred solution of the obtained solid (41.6 mg, 0.071 mmol, 1.0 e) in DCM (1.4 ml) at room temperature. After stirring for 2 h, the reaction solution was evaporated under reduced pressure, and the product was washed with Et_2_O (2 ml × 2). The crude product was then used in the next step without further purification. DIPEA (0.24 ml, 1.4 mmol, 20.0 eq) was added to a stirred solution of the obtained amine TFA salt and 5-FITC (27.8 mg, 0.071 mmol, 1.0 eq) in DMF (0.36 ml) at room temperature. After 4 h, the reaction was poured into aqueous 1 N HCl (2 ml) to precipitate the product. The solid was filtered, washed with aqueous 1 N HCl (2 ml) and purified by PTLC using chloroform and methanol (5 : 1, *v*/*v*) to give Py_3_-FITC (35.0 mg, 0.040 mmol), an orange solid obtained at a 41% yield in three steps. MeO-Py4-GABA-FITC (Py_4_-FITC) was synthesized by the same protocol as was used for producing Py_3_-FITC, and a 31% overall yield from the corresponding MeO-Py_4_-NH2 was obtained [23]. All of the reagents were purchased from Sigma-Aldrich.

### 2.5. Storage and usage of Py_3_-FITC

Py_3_-FITC is stable for over a year in a dry state at -30°C. Stock solution (10 mM in DMSO) in a dark place is stable for several months at −30°C by preventing freezing and thawing and was diluted immediately before use. It would probably cost 500 USD or less to synthesize several hundred milligrams of Py_3_-FITC using our protocol from commercially available materials.

### 2.6. Fluorescent reagent staining

Fluorescein, 5-FITC-labelled benzylamine, Py_3_-FITC and Py_4_-FITC were dissolved in DMSO to generate 10 mM stock solutions, which were stored at −30°C. Fertilized eggs were incubated in E3 solution (5 mM NaCl, 0.17 mM KCl, 0.33 mM CaCl_2_ and 0.33 mM MgSO_4_, with 0.0002% methylene blue). For early-stage staining, the chorion was removed by pronase (0.2 mg ml^−1^ in E3) treatment (28.5°C for about 7 min) at one-cell stage. For later-stage staining, the chorion was manually removed by forceps at 1 day post-fertilization (dpf). The embryos were treated with 10 µM fluorescein, 5-FITC-labelled benzylamine, Py_3_-FITC or Py_4_-FITC in E3 1 dpf. Then, 0.003% 1-phenyl-2-thiourea (PTU) (w/v) was added to E3 to prevent pigmentation. A fluorescent reagent was added to 24-well dishes and protected from light during the incubation. Immediately before the observation was performed, the fluorescent reagent-treated embryos were washed with E3 medium three times. For imaging at 24 h post-fertilization (hpf), embryos were treated with each compound for 5 h beginning at 19 hpf. Then, the embryos were anaesthetized by 0.016% (w/v) tricaine (MS-222) in E3 and mounted in 0.7–2% low-melting-temperature agarose in E3 medium with 0.016% tricaine on a glass slide using a silicon rubber ring made in-house. Z-stack images were taken by an SP8 or LSM780 confocal laser scanning microscope (Leica, Germany; Zeiss, Germany). For fixed zebrafish staining, embryos were first fixed with 4% paraformaldehyde (PFA) for 1 h. Embryos were washed and removed into acetone for 1 h (−30°C). Then embryos were stained with 10 µM Py_3_-FITC overnight.

### 2.7. Double staining with Py_3_-FITC and Alizarin Red S

The embryos that were treated with Py_3_-FITC from 1 dpf to 6 dpf, as mentioned above, were transferred into 10 µM Py_3_-FITC and 50 µM Alizarin Red S (Sigma-Aldrich) solution for 24 h and then washed with E3 medium three times. Z-stack images were taken as mentioned above.

### 2.8. Scale staining

Zebrafish (three-month post-fertilization, 3 mpf) were anaesthetized with 0.016% tricaine added to the water. The scales were picked up by forceps and treated with 10 µM Py_3_-FITC (diluted in 1 × PBS) for one hour and then washed with 1 × PBS overnight. Images were taken by a stereoscopic fluorescence microscope and digital camera (Leica M165FC and DFC7000T, Germany).

### 2.9. Antibody staining

Py_3_-FITC-treated embryos were washed with E3 medium, anaesthetized with 0.016% (w/v) tricaine (MS-222) and fixed overnight with 4% PFA at 4°C. The fixed embryos were washed twice with PBST (1 × PBS and 0.1% Triton X-100) for 5 min and treated with distilled water for 5 min. Then, the embryos were post-fixed and permeabilized with acetone for 1 h at −30°C. The embryos were washed with distilled water for 5 min and PBST (1 × PBS and 0.5% Triton X-100) twice for 30 min each time and incubated in PBST (1 × PBS and 0.5% Triton X-100) containing 1% Block Ace (DS Pharma Biomedical) for 1 h. The embryos were incubated at 4°C overnight with anti-V-ATPase subunit A (diluted 1 : 1000 with PBST containing 1% Block Ace, rabbit, #A00938-40, GenScript) antibody, anti-collagen type I (diluted 1 : 1000, mouse monoclonal; SP1. D8, DSHB) antibody and anti-collagen type II (diluted 1 : 1000, mouse monoclonal; II6B3, DSHB) antibody. Then, the embryos were washed twice with PBST (1 × PBS and 0.5% Triton X-100) for 15 min, incubated overnight with Alexa Fluor 594-conjugated anti-rabbit IgG or Rhodamine Red X anti-mouse IgG (diluted 1 : 400 with PBST containing 1% Block Ace, Jackson) at 4°C and then washed twice with PBST for 15 min each time. Images were taken with a confocal laser scanning microscope.

For the antibody staining of scales, 3 mpf zebrafish were anaesthetized with 0.016% tricaine placed in the water system, and the scales were picked up by forceps. The scales were washed with PBS and treated with 10 µM Py_3_-FITC in PBS for 1 h. The solution was removed, and the scales were washed with PBS twice and incubated overnight. The scales were fixed with 4% PFA for 1 h at 4°C. Then, the scales were incubated overnight with anti-collagen type I (diluted 1 : 100, mouse monoclonal; SP1. D8, DSHB) antibody or anti-collagen type II (diluted 1 : 100, mouse monoclonal; II6B3, DSHB) antibody at 4°C. The scales were washed with PBST (0.05% Triton X-100), incubated overnight with Rhodamine Red-X conjugated anti-mouse IgG (diluted 1 : 400, cat. no. 715-295-151, Jackson ImmunoResearch) at 4°C and washed with PBST (1 × PBS, 0.5% Triton X-100) twice for 15 min each time. Images were taken by a stereoscopic fluorescence microscope with a digital camera (Leica M165FC and DFC7000T, Germany).

### 2.10. Cell experiment

HFL-1 cells (2.0 × 10^4^ cells) were cultured in 24-well plates (DMEM supplemented with 10 mM HEPES). TGF-β1 (10 ng ml^−1^) (no. 100-21C, Peprotech) was added. Next, the cells were fixed with 4% PFA for 15 min on ice and then permeabilized and blocked in PBS containing 0.3% Triton X-100 and 3% BSA for 1 h at room temperature. The fixed cells were incubated overnight with anti-collagen type I (SP1. D8) antibody diluted with PBS containing 0.3% Triton X-100 and 1% BSA (1 : 100) at 4°C. After washing with PBS, the cells were stained with Alexa Fluor 594-conjugated anti-mouse IgG (diluted 1 : 400, no. A11005, Invitrogen). The cells were washed with PBS and incubated overnight with 10 µM Py_3_-FITC in PBS at 4°C. After washing with PBS three times, the cells were mounted with PBS containing 50% glycerol.

### 2.11. Data analysis

Z-stack confocal images were processed and analysed, and z-projection views were generated by ImageJ software (NIH). Illustrator (Adobe) and C4D (Maxon Computer) were used to construct illustrations of the opercles. The whole-view image of the embryo was composited by using Image Composite editor 2.0. (Microsoft).

## 3. Results

### 3.1. Py_3_-FITC can stain specific tissues of the zebrafish embryo

**Figure 1.**
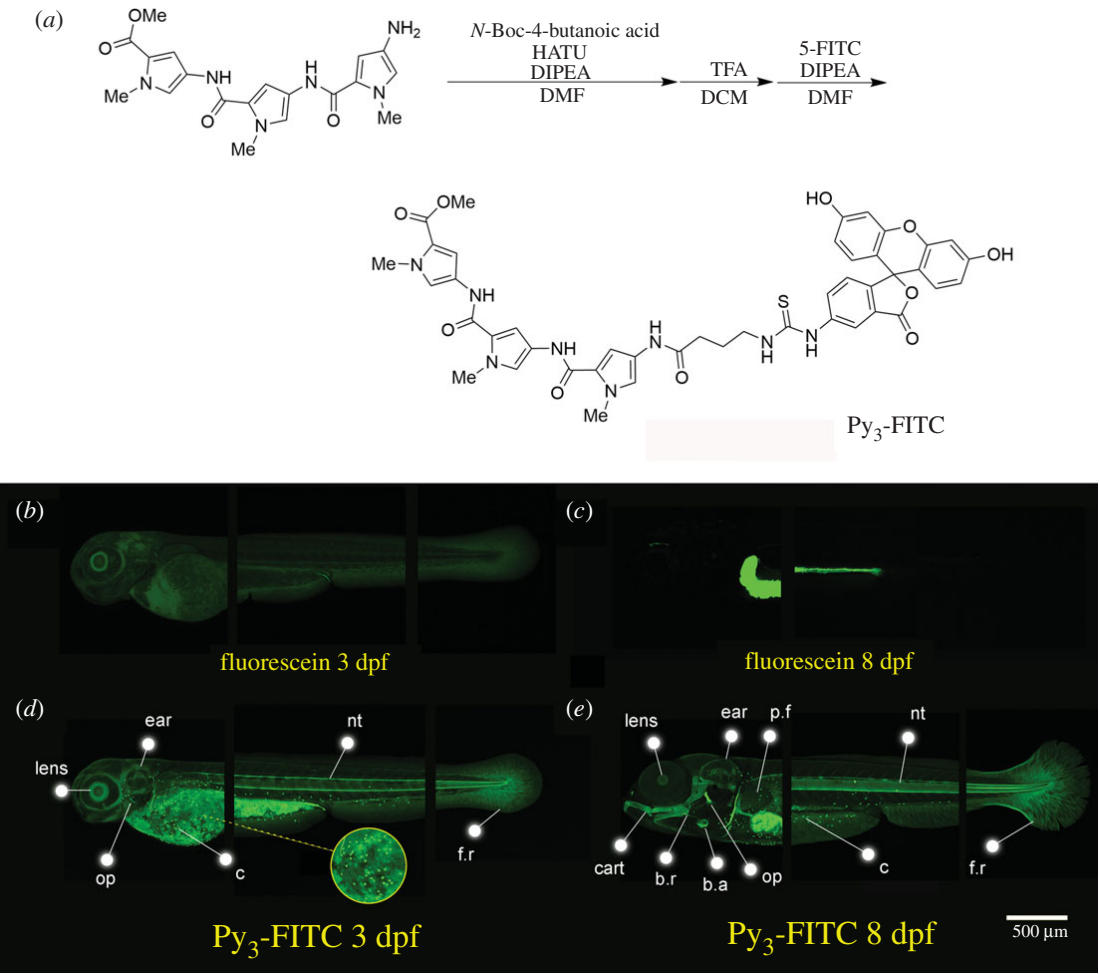
Py_3_-FITC can stain several tissues of zebrafish embryos. (*a*) Experimental procedure for the synthesis of Py_3_-FITC. Additionally, see electronic supplementary material, file S1 and figure S1. (*b*,*c*) Lateral view of fluorescein-stained zebrafish and (*d*,*e*) Py_3_-FITC-stained zebrafish embryos at (*b*,*d*) 3 dpf and (*c*,*e*) 8 dpf. Py_3_-FITC-stained lens, ear, opercle (op), notochord (nt) and cells distributed on the surface of the yolk sac and trunk region (*c*), fin rays (f.r.) 3 dpf. Jaw cartilage (cart), branchiostegal rays (b.r.), b.a. and pectoral fins (p.f.) were also stained 8 dpf. Scale bar in *e*, 500 µm.

**Figure 2.**
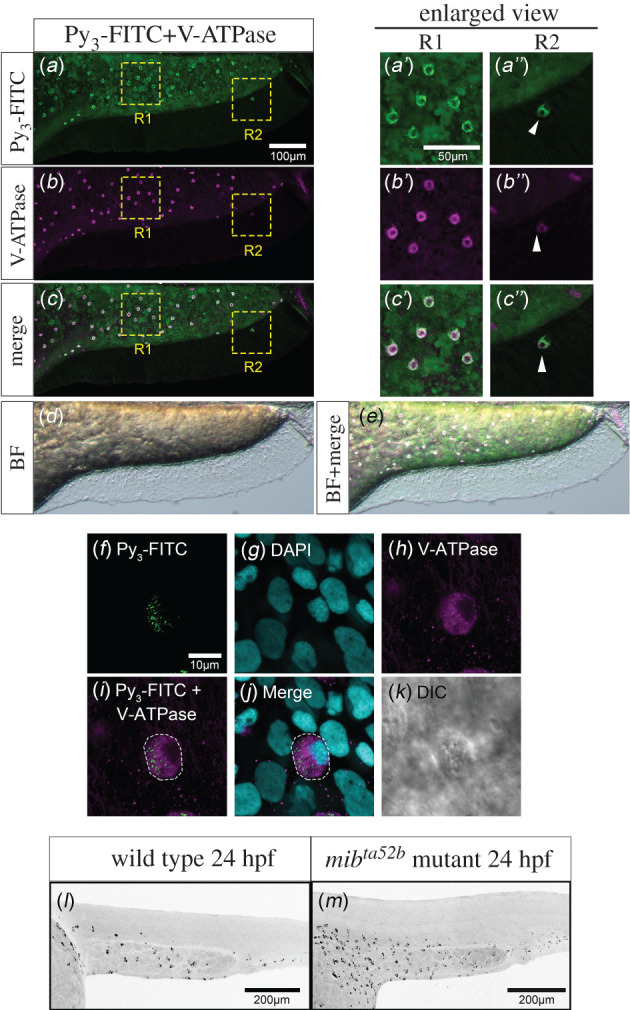
Py_3_-FITC-positive cells on the yolk and trunk surface are HR cells. Lateral views of Py_3_-FITC and anti-V-ATPase antibody double-stained zebrafish embryos at 3 dpf. (*a*) Py_3_-FITC-stained cells and (*b*) V-ATPase-expressing cells mostly overlapped (*c*). (*d*) Bright field image. (*e*) The overlaid image of *c* and *d*. Enlarged views of R1 and R2 region of Py_3_-FTIC staining (*a′, a″*), V-ATPase staining (*b′, b″*) and merged view (*c′, c″*) are also shown here. The white arrowhead indicates Py_3_-FITC-positive HR cell on the ventral fin (*a″, b″, c″*). (*f–k*) High magnification view of Py_3_-FITC-stained HR cells in 3 dpf fixed zebrafish. The projected image of Py_3_-FITC staining (*f*), the projected image of DAPI staining (*g*), the projected image of V-ATPase antibody staining (*h*), The overlaid image (*i*) of *f* and *h*, merged view (*j*) and DIC image (*k*) are shown. (*l*,*m*) Lateral views of Py_3_-FITC-stained wild-type (*l*) and *mib^ta52b^*-mutant (*m*) zebrafish embryos at 24 hpf. Embryos were treated with 10 *μ*M Py_3_-FITC for 5 h from 19 hpf (*l, m*). Py_3_-FITC-positive cells were significantly increased in Notch signalling-deficient *mib^ta52b^* embryos. Scale bars in *a*, 100 µm; *a′*, 50 µm; *f*, 10 µm; *l, m,* 200 µm.

We synthesized 5-FITC-labelled benzylamine and FITC-conjugated pyrrole polyamide trimers and tetramers, Py_3_-FITC and Py_4_-FITC, respectively (electronic supplementary material, figure S1a). Specifically, we depict the synthesis of Py_3_-FITC and its ^1^H-NMR map in detail (figure 1*a*; electronic supplementary material, figure S1b,c). We examined Py_3_-FITC staining from the 1-cell stage to the shield stage (6 hpf). We did not observe any stained cells during these early developmental stages in Py_3_-FITC-treated embryos (electronic supplementary material, figure S2a,b). Therefore, we focused on stained zebrafish embryos at later stage. We treated the zebrafish embryos from 1 dpf to 12 dpf with these compounds at a 10 µM concentration, and no obvious toxic effect was observed. When compared with fluorescein, these compounds selectively stained some body parts (figure 1*b–e*; electronic supplementary material, figure S3a–c). Compared to fluorescein and the other FITC-conjugated compounds at the same concentration, Py_3_-FITC-stained tissues more intensely (figure 1*b*–*e*; electronic supplementary material, figure S3a–c). Specific Tissues stained by Py_3_-FITC were the lens, ear, notochord (nt), jaw cartilage (cart), opercle (op), bulbus arteriosus (b.a.), pectoral fin (p.f.), branchiostegal rays (b.r.), fin rays (f.r.) and cells distributed on the surface of the yolk sac and trunk region (c) (figure 1*d,e*; electronic supplementary material, figure S4). On the other hand, Py_4_-FITC, which has one additional Py, did not stain the tissues intensely, probably because of a low uptake rate (electronic supplementary material, figure S3a–c). We also compared with zebrafish embryos stained with different concentration of Py_3_-FITC and Py_4_-FITC. The intensity of fluorescence in the 1 µM Py_3_-FITC-stained sample was much weaker than that in the 10 µM Py_3_-FITC-stained sample (electronic supplementary material, figure S5a,b). Higher concentrations of Py_3_-FITC (50, 100 µM) increased background staining but did not enhance staining compared to 10 µM Py_3_-FITC (electronic supplementary material, figure S5a–d). Higher concentrations of Py_4_-FITC (50, 100 µM) never stained the specific structure of the embryo (electronic supplementary material, figure S5e,f). In addition, higher concentrations of Py_3_-FITC (more than 50 µM) led to an increase in embryonic mortality. Although 5 h treatment (figure 2*l*,*m*) with Py_3_-FITC was sufficient to observe stained cells at 1 dpf, staining of cartilage, ear, opercle, bulbous arteriosus and notochord tissue requires at least 10 h at 4 dpf (electronic supplementary material, figure S6a–c). These results indicate that it takes more time to stain zebrafish with Py_3_-FITC at later embryonic stages. In addition, Py_3_-FITC-labelled cells and structures remained detectable for up to 5 days after washout, although the fluorescence intensity was attenuated (electronic supplementary material, figure S7a–g). We also tested direct injection of Py_3_-FITC or Py_4_-FITC at one-cell stage. However, no specific structure of the embryo could be stained by Py_3_-FITC or Py_4_-FITC injection (electronic supplementary material, figure S8a–f). Furthermore, the developmental delay or defects were observed by direct injection of Py_3_-FITC or Py_4_-FITC (electronic supplementary material, figure S8g–l).

In addition, we performed Triton X-100 treatment and 10 µM Py_3_-FITC staining after 4% PFA fixation at 4°C overnight or at room temperature for 1 h. After fixation, any specific tissues of zebrafish could not be stained with Py_3_-FITC (electronic supplementary material, figure S9a,b).

### 3.2. HR cells were stained with Py_3_-FITC

The distribution pattern of Py_3_-FITC-positive cells on the yolk sac and the trunk region is similar to that of ionocytes (figure 1*d*,*e*) [16]. Previously, ionocytes were identified in the skin of zebrafish embryos as early as 24 h after fertilization, and they consist of several types of ionocytes: HR cells, NaR cells, NCC cells, slc26 cells and KS cells [15,16,24]. NR cells are widely present in both the ventral and dorsal regions of the trunk and tail, whereas HR cells are more restricted to ventral regions [16]. Since Py_3_-FITC-positive cells were observed mostly in ventral regions, we performed double staining with V-type H+ ATPase antibody and Py_3_-FITC. We found that Py_3_-FITC-positive cells merged with the cells detected by the V-type H+ ATPase antibody, suggesting that Py_3_-FITC can selectively stain HR cells (figure 2*a*–*e*).

In addition, Py_3_-FITC staining was present neither on the membrane of HR cells nor in nucleus. An intracellular dot-like staining pattern was observed at high magnification of Py_3_-FITC and V-ATPase double staining (figure 2*f*–*k*). These data suggest that HR cells might actively be stained by Py_3_-FITC.

Notch signalling is involved in the differentiation of HR cells, and the number of HR cells is increased in the Notch signalling-deficient mutant *mind bomb^ta52b^* (*mib^ta52b^*) [25]. Therefore, we examined the numbers of Py_3_-FITC-positive cells and found that they were significantly increased in *mib^ta52b^* mutants at 24 hpf (figure 2*l*,*m*).

Together, these results suggest that Py_3_-FITC-stained cells on the surface of the yolk sac and trunk region are HR cells.

### 3.3. Cartilage can be stained with Py_3_-FITC

**Figure 3.**
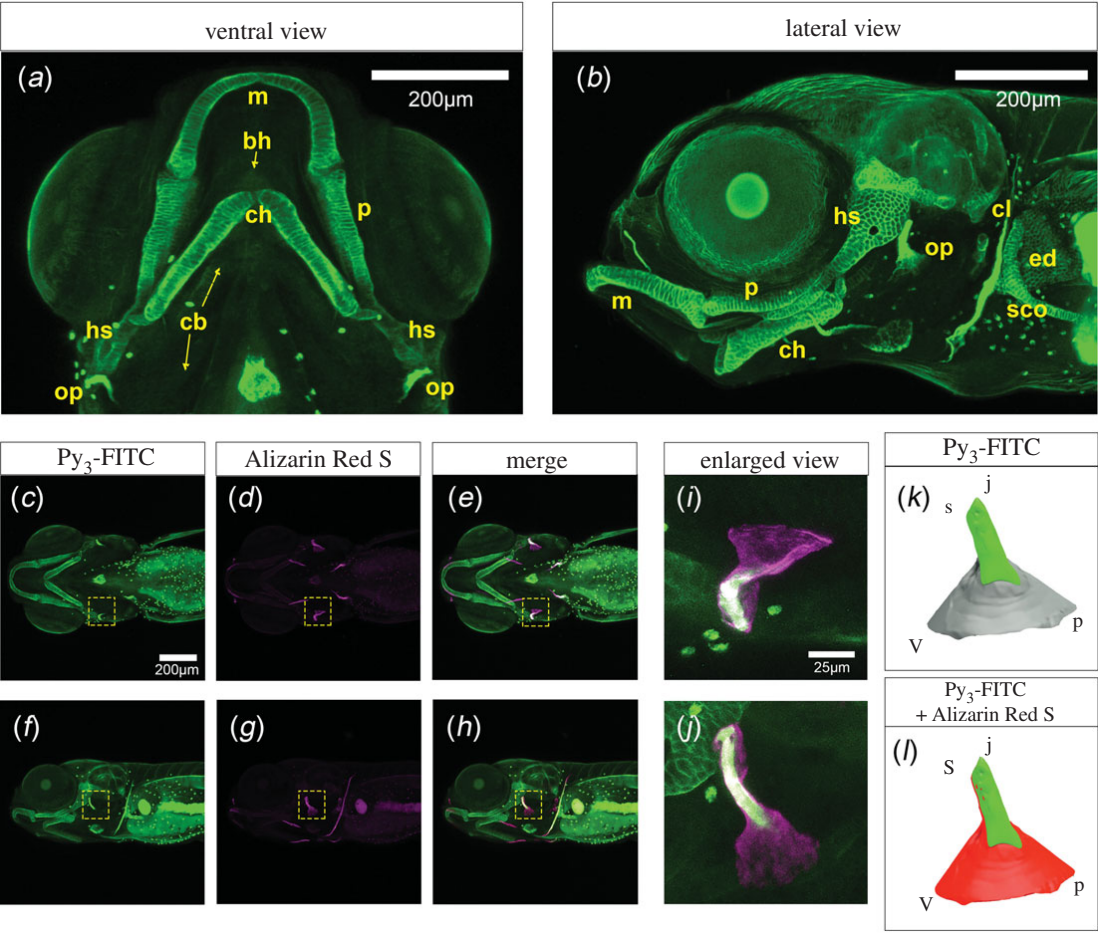
Cartilage tissues were stained with Py_3_-FITC. (*a*) Ventral and (*b*) lateral views of Py_3_-FITC-stained cartilage tissues 10 dpf. Meckel's (m), palatoquadrate (p), hyosymplectic (hs), ceratohyal (ch), scapulocoracoid (sco), cleithrum (cl), endoskeletal disc (ed) and opercle (op) were stained. (*c–e*) Ventral and (*f–h*) lateral views of Py_3_-FITC and Alizarin Red S double-stained zebrafish embryos 7 dpf. (*i,j*) Enlarged view of the yellow dotted squares shown in *e, h*. (*k,l*) Graphical illustration of Py_3_-FITC- and Alizarin Red S-stained opercles in *i* and *j*. Green indicates the Py_3_-FITC-stained region, and red indicates the Alizarin Red S-stained region of the opercles. The Py_3_-FITC-stained region was partially merged with the Alizarin Red-stained region. Parts of the opercle, joint apex (j), ventral (v), posterior (p) and joint socket (s) were visualized. Scale bars in *a, b, c*, 200 μm; *i*, 25 μm.

We next examined cartilage stained with Py_3_-FITC in the head region because Py_3_-FITC-positive regions are similar to those stained by Alcian blue [26–28]. At 10 dpf, among the Alcian blue-stained elements, Py_3_-FITC clearly stained cartilage elements, including Meckel's cartilage (m) and the palatoquadrate (p), hyosymplectic (hs), ceratohyal (ch) and opercle (op) tissues, whereas the basihyal (bh) and ceratobranchials (cb) were weakly stained (figure 3*a*,*b*). In the pectoral fin region, the scapulocoracoid (sco), cleithrum (cl) and endoskeletal disc (ed) were stained by Py_3_-FITC (figure 3*a*,*b*). Aceto *et al*. [29] showed that the basihyal and ceratobranchials are not mineralized at 10 dpf. Furthermore, Py_3_-FITC rarely stained the basihyal and ceratobranchials at 4 dpf (electronic supplementary material, figure S10a, b). These results indicate that Py_3_-FITC could not detect the basihyal and ceratobranchials, although they were not mineralized yet. Future studies should clarify how Py_3_-FITC has different sensitivities toward developing cartilage.

Taking advantage of live staining with Py_3_-FITC, compared to Alcian blue, which requires tissue fixation, we conducted double live staining using Py_3_-FITC and Alizarin Red S, which stains mineralized bone [30]. We observed distinct patterns of staining with Py_3_-FITC and Alizarin Red S, and as expected, they stained cartilage and bone, respectively (figure 3*c*–*j*). Double staining revealed the morphology of early-developing dermal bone, the opercle, which forms a plate-like rigid to support the gill [31]. Py_3_-FITC stained the upper joint region of the opercle, and Alizarin Red S stained the mineralized fan-shaped joint socket (figure 3*i*–*l*).

### 3.4. The heart chamber, the bulbus arteriosus, is stained with Py_3_-FITC

**Figure 4.**
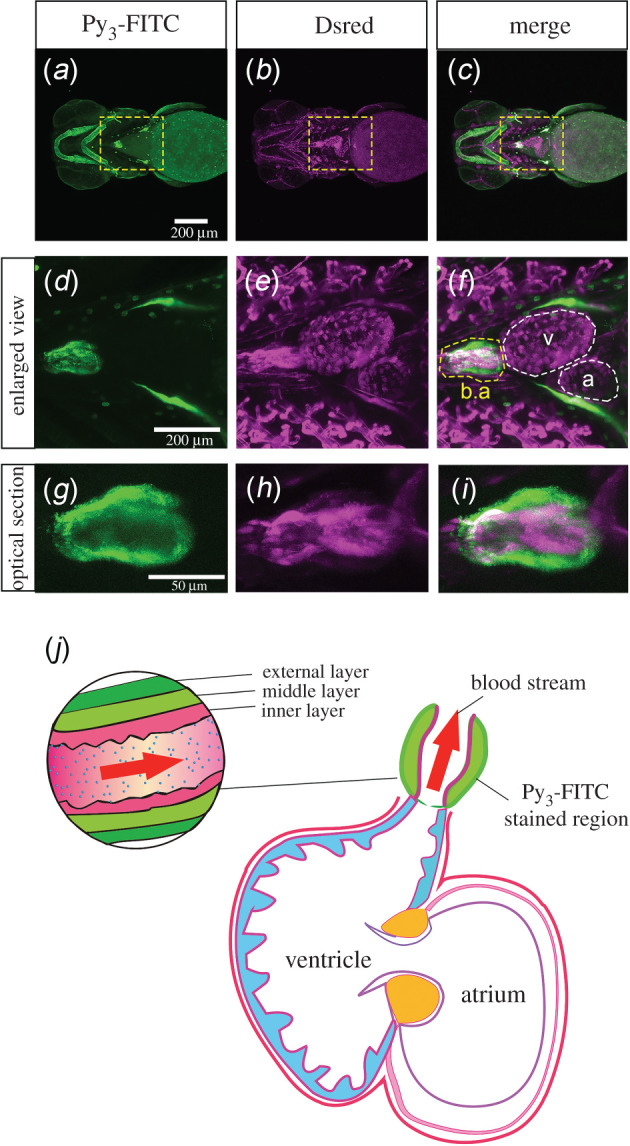
Bulbus arteriosus was stained with Py_3_-FITC. (*a–c*) Ventral view of the head and cardiomyocytes of Py_3_-FITC-stained *Tg(fli:dsRed);casper* zebrafish 7 dpf. (*d–f*) Enlarged views of the yellow boxed regions shown in *a–c*. Py_3_-FITC-stained b.a. but not the atrium (a) or ventricle (v) region. (*g–i*) Optical section views of Py_3_-FITC-stained and DsRed-positive regions in the b.a.. The graphical cross-sectional view of the zebrafish heart, which consists of the b.a., atrium and ventricle (*j*). The b.a. consists of inner and middle/external layers. Red arrows represent the direction of the bloodstream. The Py_3_-FITC-stained region is the middle/external layers of the b.a.. Scale bars in *a*, 200 µm; *g*, 50 µm.

We found that Py_3_-FITC stained the oval ball-like region of the heart after 5 dpf through 12 dpf (electronic supplementary material, figure S4a–l). In zebrafish embryos, the ventricle and atrium are formed from the heart tube in the early developmental stage. Subsequently, the b.a. is formed in the anterior region of the ventricle after 2 dpf [32]. To investigate the FITC-positive region of the heart in detail, we performed Py_3_-FITC staining of *Tg(fli:dsRed);casper*, which expresses DsRed in endothelial cells [18,19]. Time-lapse video imaging showed rhythmic signals of Py_3_-FITC and DsRed (electronic supplementary material, movie S1). Furthermore, we found that Py_3_-FITC stained outside of the DsRed area in the b.a. (figure 4*a*–*j*). The b.a. is a unique elastic structure in fish that functions to dampen the pressure pulse generated by the ventricle as a form of circulatory adjustment [33]. The external layer of the b.a. is known to be formed by numerous cell types embedded in a collagenous matrix [33]. Considering that Py_3_-FITC stained the cartilage, notochord and fin rays, which are also collagen-rich tissues, Py_3_-FITC may have a high affinity for the collagen-rich matrix.

### 3.5. Py_3_-FITC stains collagen-rich tissues

**Figure 5.**
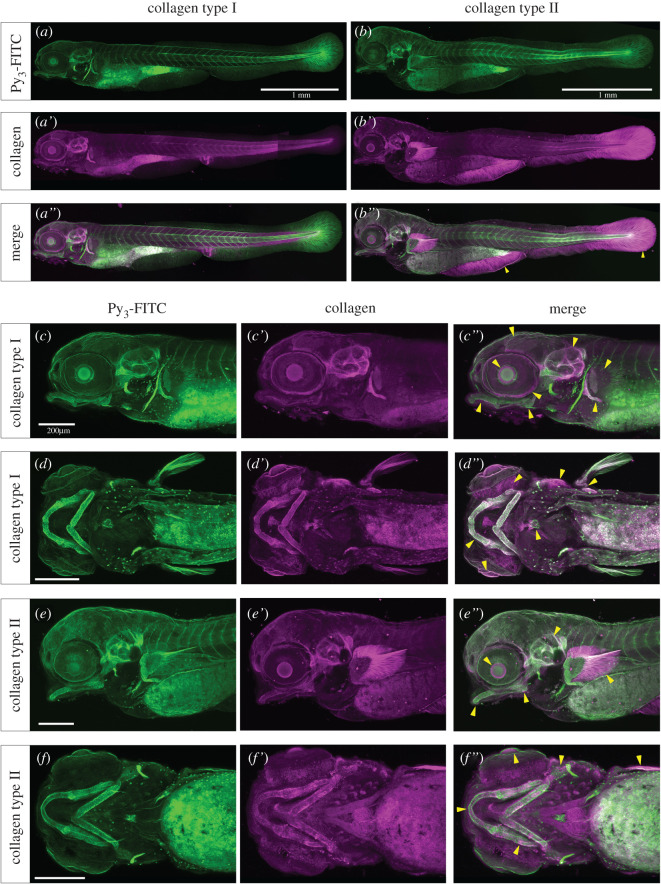
Py3-FITC can stain collagen type I- or II-positive tissues in zebrafish embryos. Reconstructed (*a*–*b*″,*c*–*c*″,*e*–*e*″) lateral views and (*d*–*d*″,*f*–*f*″) ventral views of the Py3-FITC and anti-collagen (*a*–*a*″,*c*–*d*″) type I and (*b*–*b*″,*e*–*f*″) type II antibody-stained zebrafish embryos at 5 dpf. (*a*″,*b*″,*c*″,*d*″,*e*″,*f*″) Py3-FITC-stained regions overlapped with collagen type I- or type II-positive regions. The yellow arrowheads represent Py3-FITC and collagen expression overlapping regions (*b*″,*c*″,*d*″,*e*″,*f*″). Enlarged views are shown of collagen (*c*′,*d*′) type I- and (*e*′,*f*′) type II-stained regions and their merged images (*c*″,*d*″,*e*″,*f*″). Scale bars in *a*,*b*, 1 mm; *c*,*d*,*e*,*f*, 200 μm.

We next compared regions stained by Py_3_-FITC with those stained by anti-collagen type I or anti-collagen type II antibody. Most of the tissues stained by anti-collagen type I and II antibodies were also stained positively by Py_3_-FITC (figure 5*a*,*b*″). The notochord was stained with Py_3_-FITC, which was consistent with both anti-collagen type I and II antibodies positive region (electronic supplementary material, figure S11a–d″). Collagen type I-stained areas overlapping with Py_3_-FITC-stained areas were mainly located in the ear, eye, jaw skeletal elements (m, ch and p), fin elements (sco and ed), b.a., and other areas, as shown in figure 5*c*,*d*″. On the other hand, the collagen type II-stained areas overlapping with Py_3_-FITC-stained areas were mainly observed in jaw skeletal elements and the tail fin ray region (figure 5*b*-*b*″, *e*-*f*″). However, not all collagen I- and II-positive areas overlapped with the Py_3_-FITC-positive areas (figure 5*a*–*f*″). Additionally, we examined whether Py_3_-FITC-stained adult scales since the zebrafish scale contains collagen type I [34]. We found that most of the collagen type I-positive areas were also Py_3_-FITC positive, and collagen type II was not detected in the scales (electronic supplementary material, figure S11e-f″).

**Figure 6.**
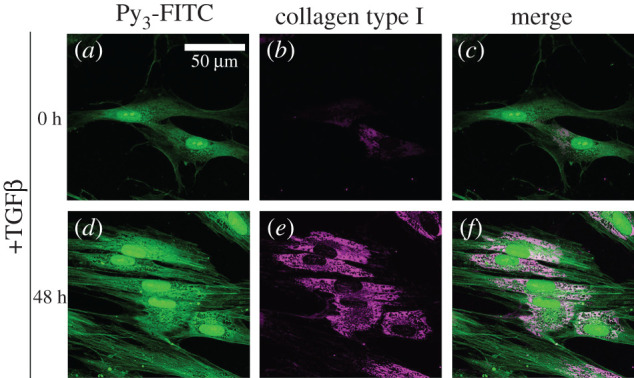
Py_3_-FITC can be used to detect TGF-β1-induced collagen type I. HFL-1 cells were treated with TGF-β1 for 0 h (*a–c*) or 48 h (*d–f*) and then stained with Py_3_-FITC (*a*,*d*) and collagen type I antibody (*b*,*e*), and their merged images are shown (*c*,*f*). Collagen type I expression was increased by 48 h of TGF-β1 treatment. The cytoplasmic signal of Py_3_-FITC was also increased by TGF-β1 and was partially merged with that of collagen type I (*c*,*f*). Scale bar in *a*, 50 μm.

Furthermore, we examined the relationship between Py_3_-FITC and collagen using cultured human cells. In HFL-1 cells, transforming growth factor-β1 (TGF-β1) stimulates collagen type I expression [35]. Thus, we performed double staining with anti-collagen type I antibody and Py_3_-FITC in HFL-1 cells treated with or without TGF-β1. We observed that Py_3_-FITC staining was increased in the cytoplasm, as collagen type I accumulated in the cytoplasm after TGF-β1 stimulation (figure 6*a*–*f*). We also performed Py_3_-FITC staining in living HFL-1 cells. Nuclei of living HFL-1 cells could not be stained by Py_3_-FITC clearly (electronic supplementary material, figure S12a, b), and the signal intensity was weaker compared to that in fixed samples. Therefore, the permeabilization step after fixation may be important for Py_3_-FITC staining in cultured cells. Moreover, we did not observe obvious staining with 5-FITC-labelled benzylamine, while Py_4_-FITC showed strong signals in the cell nucleus for both living cells or fixed cells (electronic supplementary material, figure S13a–h). However, neither 5-FITC-labelled benzylamine nor Py_4_-FITC showed an increase in their cytosolic accumulation in response to TGF-β1 stimulation, as was observed for Py_3_-FITC (electronic supplementary material, figure S13a–h).

Taken together, these *in vivo* and *in vitro* results suggest that Py_3_-FITC can be used to detect collagen-rich fibrous tissues.

## 4. Discussion

### 4.1. Py3-FITC is a new low-molecular-weight fluorescent probe for collagen-rich tissues and others

Several fluorescent probes for collagen detection have been developed thus far [11–13]. However, to our knowledge, previous probes have not yet been used for detecting collagen in living animals. Transgenic lines in which fluorescence-fused collagen protein is expressed have also been reported [14]. However, in these transgenic lines, the collagen subtypes detectable are limited.

Our study revealed that Py_3_-FITC, a low-molecular-weight compound consisting of FITC-conjugated pyrrole trimers, might stain several types of collagen. Consistently, in HFL-1 cells, the Py_3_-FITC signal was only partially merged with that of collagen type I. Collagen type III and type V are also induced by TGF-β1 stimulation in HFL-1 cells [36,37]. Therefore, an increase in other collagens, such as type III and type V, also contributes to an increase in Py_3_-FITC-positive components in TGF-β1-stimulated HFL-1 cells.

Collagen forms a right-handed triple helix [7]. Py_3_-FITC might recognize and interact with this collagen three-dimensional structure. The number of pyrrole repeats may be critical for the interactions with collagen because Py_4_-FITC, which has only one additional pyrrole, rarely stained collagen-rich tissues. For wider applications, we created other colour fluorophore-conjugated Py_3_-based compounds, such as Py_3_-Cy3 and Py_3_-rhodamine. However, these red fluorophore-conjugated Py_3_ compounds did not stain the same tissue stained by Py_3_-FITC. These results indicate that the chemical properties of fluorescein might also contribute to the selectivity of Py_3_-FITC staining. In addition, poly-Py-based polyamides are known to be able to interact with the minor groove of the DNA duplex. Fluorophore-conjugated poly-Py-based polyamides, which are expected to localize to nuclei, have been reported [38]. By contrast, some dye conjugates localize mainly in the cytoplasm, not the nucleus, suggesting that localization in the cell depends on the cellular uptake rate and cell type [3,39].

We showed that Py_3_-FITC can localize to nuclei in HFL-1 lung fibroblast cells but rarely in living zebrafish embryos. Moreover, fixed embryo nuclei were not stained by Py_3_-FITC. This indicates that fixation may be necessary but not sufficient for nuclear staining in zebrafish embryos with Py_3_-FITC. Further studies are needed to clarify these discrepancies.

### 4.2. Py_3_-FITC is a useful tool for live imaging

Py_3_-FITC induces low toxicity and therefore is useful for applications in the fields using live *in vivo* imaging. Three type tissues were stained in living organisms in this study: cartilage, notochord and scale; heart; and ionocytes.

*Cartilage, notochord and scale*. Alcian blue and Alizarin Red are two colour stains that are often used to estimate bone formation. However, double staining requires cell fixation and involves multiple staining steps [40]. Py_3_-FITC and Alizarin Red S double staining is a new and easy live imaging method for detecting bone formation in zebrafish embryos. A caveat for using this staining method is that the Py_3_-FITC staining pattern is different from the Alcian blue staining pattern to some extent. For instance, ceratobranchial cartilage is clearly stained by Alcian blue staining but not by Py_3_-FITC. By contrast, fin rays are stained by Py_3_-FITC but not by Alcian blue [40]. These differences might be due to the difference in their targets, namely, Alcian blue stains acidic mucopolysaccharides [41] and Py_3_-FITC may interact with collagen-rich tissues.

Dupret *et al*. showed that craniofacial cartilage is Alcian blue-negative at 2 dpf [42], but it could be stained with Alcian blue at 3 dpf [43]. These previous results indicate that the earliest emergence of cartilage corresponds to the first detection of Py_3_-FITC signal. In addition, the notochord could be stained with anti-collagen II antibody at 24 hpf [44]. Py_3_-FITC can detect the notochord structure at 24 hpf or even earlier stages. These results suggest that Py_3_-FITC staining can be detected at the earliest emergence of cartilage and notochord during development.

Py_3_-FITC can stain adult outer tissue, such as scales. However, new methods might be needed to deliver Py_3_-FITC into deep tissues to visualize cartilage in later-stage living juvenile or adult fish.

*Heart*. Py_3_-FITC can visualize the beating of the b.a. The b.a. is a collagen-rich compartment of the fish heart and functions as a modulator to provide steady blood to gills [45]. It has been reported that WGA lectin also stains b.a. mesenchyme at 72 hpf. However, WGA lectin is a protein and not suitable for use as a live imaging tool. By taking advantage of Py_3_-FITC, cardiac beating and rhythm in live zebrafish can be recorded.

*Ionocytes*. By contrast to the above two tissues, HR cells are not collagen-rich. Py_3_-FITC stains only HR cells and can be used in live samples. Py_3_-FITC did not stain the surface of the HR cells. Confocal analysis showed many intracellular organelles stained with Py_3_-FITC. However, the cell types with Py_3_-FITC-positive intracellular organelles and the extent to which Py_3_-FITC uptake is linked to HR cells function are currently unknown. Ionocyte analogues are also present in mammals, such as renal proximal tubular cells [15], and Py_3_-FITC may be used to help detect and study them. These issues remain to be addressed in future research.

## 5. Conclusion

We generated a new fluorophore-conjugated polypyrrole, Py_3_-FITC. In living zebrafish embryos, Py_3_-FITC can detect collagen-rich tissues such as cartilage, b.a., notochord, fin ray, etc. Py_3_-FITC also detects HR cells, which are one type of ionocytes. It has low toxicity, and the staining method is rapid and easy to use. Py_3_-FITC is a useful live imaging tool for detecting collagen-rich tissue morphogenesis and HR cells distribution. Collagen is related to wound healing and fibrosis. Therefore, with modification, Py_3_-FITC might be used as a new diagnostic or therapeutic agent.

## Data accessibility

This article has no additional accessible data.
